# Risk of Eating Disorders and Social Desirability among Higher Education Students: Comparison of Nutrition Students with Other Courses

**DOI:** 10.3390/healthcare12070744

**Published:** 2024-03-29

**Authors:** Sandra Fernandes, Carolina Costa, Ingrid Sayumi Nakamura, Rui Poínhos, Bruno M. P. M. Oliveira

**Affiliations:** 1 Faculty of Nutrition and Food Sciences, University of Porto, Rua do Campo Alegre, 823, 4150-180 Porto, Portugal; sandra-abreu-f@hotmail.com (S.F.); cnrcosta@gmail.com (C.C.); nakamura.ingrid@gmail.com (I.S.N.); ruipoinhos@fcna.up.pt (R.P.); 2 Laboratory of Artificial Intelligence and Decision Support, Institute for Systems and Computer Engineering—Technology and Science, University of Porto, 4200-465 Porto, Portugal

**Keywords:** risk of eating disorders, EAT-26, social desirability, nutrition students, higher education students

## Abstract

The transition to college is a period of higher risk of the development of eating disorders, with nutrition/dietetics students representing a group of particular vulnerability. Hence, it is interesting to assess eating disorders, taking into consideration potential sources of bias, including social desirability. Our aims were to compare the risk of eating disorders between students of nutrition/dietetics and those attending other courses and to study potential social desirability biases. A total of 799 higher education students (81.7% females) aged 18 to 27 years old completed a questionnaire assessing the risk of eating disorders (EAT-26) and social desirability (composite version of the Marlowe–Crowne Social Desirability Scale). The proportion of students with a high risk of eating disorders was higher among females (14.5% vs. 8.2%, *p* = 0.044). Nutrition/dietetics students did not differ from those attending other courses regarding the risk of eating disorders. The social desirability bias when assessing the risk of eating disorders was overall low (EAT-26 total score: r = −0.080, *p* = 0.024). Social desirability correlated negatively with the Diet (r = −0.129, *p* < 0.001) and Bulimia and food preoccupation subscales (r = −0.180, *p* < 0.001) and positively with Oral self-control (r = 0.139, *p* < 0.001).

## 1. Introduction

Eating behavior refers to motivations and cognitive processes related to the selection and decision of which foods to eat, and it may be influenced by several factors, including psychological and sociocultural ones [1,2]. Eating behavior can be analyzed by studying its dimensions, with emotional and external eating (i.e., eating in response to emotions or to external food-related cues, respectively), binge eating, dietary restraint (flexible control and rigid control), and eating self-efficacy having shown clinical relevance, or focusing on the risk and occurrence of eating disorders [1,3,4]. Eating disorders are characterized by changes in eating-related behaviors significantly compromising physical health and/or psychosocial functioning and present a multifactorial etiology [4,5].

The transition to higher education is a period of increased susceptibility to the development of eating disorders, since individuals experience psychological, physiological, and sociocultural changes that may result in a reorientation of eating behavior [6]. Nutrition and dietetics students may represent a group with specific vulnerabilities to eating disorders [7]. When analyzing risk factors for the development of eating disorders, studies have found that constant contact with food [8,9], as well as knowledge on food-related issues, weight control, and body composition [7] and the imposition of strict aesthetic standards on students, with the belief that a good appearance can be important for professional success [8,9], can result in an increased prevalence of eating disorders among nutrition/dietetics students. Also, when analyzing the motivation to attend a nutrition or dietetics course, some authors suggest the possible influence of personal experiences regarding food and weight control [10,11,12]. Indeed, some research has shown that the risk of eating disorders is higher among students in these areas compared to students on other courses [13,14]. However, some authors have found similar results but without statistical significance [8,11], and other studies have not found such differences [7,15,16,17,18]. This controversy of results in the literature may be due to several factors, namely, different environments and the use of different methodologies and/or instruments. However, assessing the risk of eating disorders among students with frequent contact with issues related to food and body image, particularly nutrition and dietetics students, is important, because it may have implications for the professional practice of these future health professionals.

In psychological research involving self-administered questionnaires, social desirability may compromise its validity as a potential source of bias [3,19,20,21]. Social desirability is defined as the individual’s tendency to convey a culturally accepted image, according to social norms, to avoid negative opinions towards socially undesirable behaviors [22,23]. The assessment of eating behavior among students or professionals in the area of nutrition or dietetics may be more prone to the effects of social desirability, since, in addition to being concerned with current aesthetic standards, these respondents may consider that knowledge in the area should be reflected in what is normative eating behavior [3]. Understanding the effects of social desirability on eating behavior may contribute to better accuracy of its assessment [24]. Therefore, this construct should not be neglected in scientific research [3,19,20,21].

To the best of our knowledge, no prior studies have considered social desirability when assessing the risk of eating disorders among nutrition/dietetic students or professionals. However, studies evaluating the relationship of this construct with dimensions of eating behavior in higher education students found negative associations with emotional [3,21,24], external [3,21], and binge eating [3,21,24] and a positive association with eating self-efficacy [3,21]. However, other studies have not found an association between social desirability and emotional [25] or binge eating [26].

In this context, the main aim of this study was to assess the risk of eating disorders among higher education students, to compare the risk of eating disorders between students of nutrition/dietetics and those attending other courses, and to analyze the effect of social desirability on the assessment of eating disorders in these students. We also aimed to study the relationships of sex, age, body mass index (BMI), and course’s year of attendance with the risk of eating disorders.

## 2. Methodology

### 2.1. Sample and Procedures

This study is part of the project “Eating behavior and risk of eating disorders in higher education students: a national longitudinal study. Comparison of nutrition/dietetics students with other courses”, approved by the Ethics Committee of the Faculty of Nutrition and Food Sciences of the University of Porto (Reference: 16/2020/CEFCNAUP/2020). It was carried out in a sample of students attending undergraduate or integrated master’s degrees at Portuguese public or private higher education institutions (universities or polytechnics). The inclusion criteria were being between 18 and 27 years old, and that the student could exercise a free and informed decision to participate in the study. Students older than 27 years were not included to reduce sociodemographic heterogeneity. Students attending higher technical and professional courses were also excluded.

All Portuguese public and private universities (n = 10) and polytechnics (n = 7) with a degree in nutrition/dietetics (Nutrition Sciences; Dietetics; Nutrition and Dietetics) were contacted. In a second phase, the faculties (n = 36), schools (n = 37), and institutes (n = 20) of those universities and polytechnics were also contacted. Nine faculties, eleven schools, three institutes, four universities, and two polytechnics agreed to participate in the research.

Data were collected between March and June 2022 through an online questionnaire sent by the institutions to the students’ institutional contacts. At the beginning of the questionnaire, the scope and purpose of the research were mentioned, and the informed consent of potential participants was requested.

The first part of the questionnaire referred to sociodemographic (sex, age) and academic characteristics (institution, course attended, academic year), as well as self-reported weight and height and desired weight. Prior diagnosis of eating disorders was also questioned. The second part of the questionnaire integrated the instruments for assessing the risk of eating disorders and social desirability.

A total of 821 students answered the questionnaire. Data from 22 participants were not analyzed due to incomplete data, considering as criterion the lack of response to more than one question per scale on the Eating Attitudes Test-26 (EAT-26) [27] or on the social desirability scale. Two multivariate outliers were also excluded. Therefore, data from 799 students were analyzed.

For comparison purposes, students were divided into three groups according to their areas of study: nutrition and dietetics (n = 110), other areas of human health (n = 202) (which include degrees or master’s in psychology, medicine, dentistry, nursing, speech therapy, physiotherapy, pharmaceutical sciences, and sports), and other non-health-related areas (n = 487).

### 2.2. Measures

The risk of eating disorders was assessed through the Portuguese version of the EAT-26 [28,29]. It consists of 26 items, organized into three subscales: Diet (e.g., “I eat diet food”), which corresponds to a pathological refusal of high-energy foods and to an intense concern with physical shape (13 items; score range: 0 to 39 points); Bulimia and food preoccupation (e.g., “I have eaten uncontrollably and felt like I couldn’t stop”), which evidences episodes of compulsive food intake followed by vomiting and/or other behaviors to avoid weight gain (6 items; score range: 0 to 18 points); and Oral self-control (e.g., “I avoid eating when I am hungry”), which reflects self-control regarding food and possible social pressures that stimulate food intake (7 items; score range: 0 to 21 points). Each item can be answered on a 6-point Likert scale (from “Always” to “Never”), with each response coded with values between 0 and 3. The total score results from the sum of the answers to all items and may range from 0 to 78 points, with total scores of 21 points or above indicating a high risk of eating disorders [29].

To assess social desirability, the composite Portuguese version of the Marlowe–Crowne Social Desirability Scale (MC-SDS) [30,31] was used. This scale includes 13 items which should be rated as true or false by the respondent. Some items correspond to sentences that describe socially desirable but uncommon behaviors (scored if answered “true”), while others describe highly common but socially undesirable behaviors (scored when answered “false”). Higher scores reflect a tendency to give more socially desirable responses [30,31].

BMI was calculated from weight (kg) divided by the square of height (m^2^). Current BMI was calculated from self-reported values, being subsequently corrected by the equation developed by Pinhão et al. (2014) [32], which predicts the actual BMI from the reported BMI, age, and sex (“adjusted BMI”), and classified according to the criteria of the World Health Organization for adults [33]. The BMI for desired weight (“desired BMI”) was also calculated. The difference between the desired and current BMI (current BMI minus desired BMI) was calculated and named “wished BMI change”.

### 2.3. Statistical Analysis

Multivariate outliers from the original data were detected after computing the Mahalanobis distance and checking if the *p*-value for the chi-square distribution with 11 degrees of freedom was *p* < 0.001. The 11 degrees of freedom correspond to the 11 variables in the MANOVA and UniANOVA procedures.

For the descriptive analysis, absolute (n) and relative (%) frequencies were calculated to summarize qualitative variables. The results of the EAT-26 scale and subscales, age, BMI, and social desirability are expressed as mean and standard deviations (SD).

The normality of the distributions of the quantitative variables was assessed using skewness and kurtosis. When the variables had a non-normal distribution, a 2-parameter Box–Cox transformation was applied together with a linear transformation, so that the median was not altered. The variables adjusted BMI, desired BMI, and wished BMI change were transformed with the exponent parameter λ = −1, and for the four EAT variables, the exponent λ = 0 was used. The transformed variables were used in inferential analysis, but descriptive statistics are presented for the untransformed variables for clarity.

We used Fisher’s exact test to assess the degree of association between (a) elevated risk of eating disorders and sex; (b) elevated risk of eating disorders and prior diagnosis of eating disorder; and (c) sex and course. The difference between sexes regarding age, BMI variables, the EAT-26 scale and subscales, and social desirability was determined using independent samples Student’s *t* test, whereas for the comparison between courses, one-way ANOVA was used. We assessed the degree of association between the EAT scale and subscales and the variables: academic year, age, adjusted BMI, wished BMI change, and social desirability using Pearson’s correlation coefficient. In addition, we performed a multivariate analysis (MANOVA and UniANOVA) to study the effects of sex, course, academic year, age, adjusted BMI, wished BMI change, and social desirability on the EAT scale and subscales. The effect size was quantified using partial eta squared (*η_p_^2^*).

Statistical treatment was performed in IBM SPSS version 27.0 for Windows. *p* values below 0.05 were considered statistically significant. Considering a statistical power of 80%, a correlation of 0.099 is likely to be significant with a sample size of 799 [34].

## 3. Results

The sample was predominantly female (81.7%, n = 653), had a mean age of 21.2 years (SD = 2.5), and 73.5% (n = 587) of students were classified as having normal weight after BMI adjustment. Of the respondents, 9.4% (n = 75) had a previous diagnosis of eating behavior disorder, with no statistically significant differences between sexes being observed (10.4% of females vs. 4.8% of males; *p* = 0.085). Regarding the risk of eating disorders, 13.4% (n = 107) were at a high risk (EAT-26 total score ≥ 21 points), with this proportion being higher among females (14.5% of females vs. 8.2% of males, *p* = 0.044). Among the respondents with previous diagnosis of eating disorders, 53.3% were currently at a high risk of eating behavior disorders, while among those without previous diagnosis, only 8.5% were at risk (*p* < 0.001).

Table 1 describes and compares age, BMI, EAT-26 total and subscales, and social desirability between sexes. Male participants were significantly older than females and presented higher adjusted and desired BMI values and lower wished BMI change. Women had higher mean scores on the EAT-26 total scale and on the three subscales, as well as on the social desirability scale, although no statistically significant differences were found.

The distribution of participants according to sex differed significantly between courses (*p* < 0.001), with nutrition and dietetics presenting a lower proportion of males (7.3%) than other areas of human health (15.3%) and other non-health-related areas (22.0%). Students attending different areas did not significantly differ regarding age, BMI variables, risk of eating disorders (EAT-26 total and subscales), or social desirability level (Table 2).

The correlations between the EAT-26 total score and subscales, age, BMI variables, and social desirability are presented in Table 3. The overall risk of eating disorders (EAT-26 total score) significantly correlates with its three subscales, being the strongest association with Diet and the weakest with Oral self-control. The Diet and the Bulimia and food preoccupation subscales presented a moderate positive correlation, while the associations with Oral self-control were very weak, and for Bulimia and food preoccupation, it was non-significant. Older participants had a lower risk of eating disorders, specifically on the dimension Oral self-control, although these associations were very weak. On the other hand, a higher adjusted BMI and wished BMI change were positively associated with a higher risk of eating disorders on the Diet and Bulimia and food preoccupation dimensions, but with a lower risk concerning Oral self-control. Social desirability was associated with lower Diet and Bulimia and food preoccupation scores, as well as a lower EAT-26 total score, while the correlation with Oral self-control was positive. Also, participants with a higher BMI and higher wished BMI change presented higher social desirability levels.

Table 4 shows the effects of sex, age, course, academic year, BMI, and social desirability on the EAT-26 scale and subscales. The independent variables significantly explained the total score in EAT-26 (*η_p_*^2^ = 0.063, *p* < 0.001), but the corrected models’ effect sizes were higher for the three subscales (*η_p_*^2^ between 0.104 and 0.180, *p* < 0.001). All significant effects had the same direction as the relationships that were found in the bivariate analysis. Wished BMI change significantly explained the overall risk of eating disorders and the scores on the three subscales and had the highest effect size in all except for Oral self-control. Social desirability presented significant effects on those three subscales, but not on the EAT-26 total score. The Bulimia and food preoccupation subscale was also explained by age and the Oral self-control subscale by adjusted BMI.

## 4. Discussion

The main aim of this study was to assess the risk of eating disorders among higher education students of nutrition/dietetics and other areas and to analyze the effect of social desirability on the assessment of eating disorders among these students. Prior research shows differences in eating behavior between sexes [3,35], so a first analysis was to study if such differences were also found for the risk of eating disorders. Our study found no statistically significant differences between sexes in the mean score of the EAT-26 and its subscales. This result is in line with a previous study [36], but in the study by Yu et al. (2018) [37], female college students scored significantly higher than their male counterparts on two EAT-26 subscales (Diet and Bulimia and food preoccupation), and in another work, this difference was found for the total EAT-26 score [35].

Concerning social desirability, we found no statistically significant differences between sexes. Other authors, who used the 33-item Marlowe–Crowne Social Desirability Scale, support this result [21,24]. However, Freitas et al. (2017) found that female nutrition students had higher levels of social desirability compared to males (14.8 vs. 17.3; *p* = 0.028) [3]. Women presented lower adjusted BMIs and also lower desired BMIs compared to men, which is in line with the results obtained by Poínhos et al. (2013) [38].

Among our sample, 13.4% of the participants were at a high risk of eating disorders (EAT-26 total score ≥ 21 points), and this proportion was higher among females (14.5% vs. 8.2%). With a slightly different cut-off point (risk ≥ 20 points), Yu et al. (2018) [37] also found a higher prevalence of eating disorder risk among female college students. On the other hand, another study among young adults did not find sex differences in the prevalence of risk (19.4% vs. 19.3%, *p* > 0.05) [35]. It is also worth noticing that the proportion of people who are at a high risk of eating disorders seems to be quite variable, with Yu and Tan (2016) [18] describing a prevalence of 10% among nutrition college students and Meulemans et al. (2014) [39] describing a prevalence of 8% in a Seventh-day Adventist higher education institution, also considering ≥20 to be the cut-off.

Regarding the associations between the three EAT-26 subscales, in our sample, the Diet subscale was positively correlated with Bulimia and food preoccupation and Oral self-control, while no significant association was found between the Bulimia and food preoccupation and Oral self-control subscales. Berland et al. (1986) [40] found a positive and statistically significant correlation between the Diet subscale and the subscales Bulimia and food preoccupation and Oral self-control, while the negative correlation between the subscales Bulimia and food preoccupation and Oral self-control was not statistically significant. According to the authors, the questions on the subscales Bulimia and food preoccupation and Oral self-control are orthogonal and seem to be referring to unrelated issues. However, Thomas et al. (2017) [41] found statistically significant correlations between the three subscales of the EAT-26, indicating that its structure may be culture-dependent.

As previously mentioned, some authors argue that the constant contact with food [8,9]; the knowledge related to food, weight control, and body composition [7]; and the belief that a good appearance may be important for professional success [8,9] may lead to an increased prevalence of eating disorders among nutrition students. In addition, some authors suggest that there may be an influence of personal experiences regarding eating and weight control when selecting a nutrition or dietetics course [10,11,12]. Taking these effects into account, there would be a tendency for a higher risk of eating disorders among nutrition/dietetics students. However, no difference or main effect of the attended course was found for the EAT-26 scale or subscales. Yu and Tan (2016) [18], with a similar grouping of students to the one that we used, found no significant differences between courses on the EAT-26 score, neither on its three dimensions, which is in line with our results. However, a study conducted only with first-year female students in Nutrition, Physical Education, Advertising and Publicity, and Business Administration found statistically significant differences, with health students showing higher scores compared to students from other areas (16.6 vs. 12.5, *p* = 0.006). Additionally, the analysis revealed that nutrition students presented the highest scores on the EAT-26, being statistically different from the scores of those attending Advertising (18.4 vs. 12.7, *p* < 0.05) and Administration courses (18.4 vs. 12.3, *p* < 0.05), but not from Physical Education students (18.4 vs. 15.3, *p* > 0.05) [14]. A study conducted with female students of Nutrition, Nursing, and Biological Sciences did not show statistically significant differences among courses. However, the results suggest a higher probability of female nutrition students developing eating disorders (23.8% vs. 9.8% vs. 7.7%, considering ≥ 21 as cut-off) [42]. Another study supports these results, as it showed that students attending Nutrition courses were more exposed to the risk of developing eating disorders than those from other health courses (β = 0.10, *p* = 0.03) [36].

The controversy of results found in the literature, both in the presence or absence of significant differences and in terms of the proportion of participants at a high risk, may result from the criteria used by the studies, such as the age range, courses, and academic years included, or from different environments. The grouping of students according to courses, as well as the different subsample sizes, may also contribute to the discrepancy of results. In addition, the literature presents different cut-off points in the EAT-26 to define the risk of eating disorders.

Another goal of our study was to analyze the effect of the course year on the risk of eating disorders. While negative very weak correlations were found between the academic year and the EAT-26 total and Diet subscale, no significant effect of year was found in the multivariate analysis. Our results are not in line with those from a study among students attending health courses, in which the authors found a weak but positive and significant association between the academic year and the EAT-26 score [36].

The adjusted BMI and the wished BMI change correlated positively with each other and with the EAT-26 and Diet and Bulimia and food preoccupation subscales and negatively with Oral self-control. However, only the wished BMI change significantly explained the EAT-26 and its three subscales, with a greater effect on the Diet subscale. Multivariate analysis only confirmed the negative correlation between the adjusted BMI and Oral self-control. A peculiarity in the results is regarding the negative correlations with the Oral self-control dimension: the higher the adjusted BMI and the wished BMI change were, the lower their Oral self-control related to food and possible social pressures that encourage food intake.

Negative correlations were found between social desirability and the adjusted BMI, wished BMI change, EAT-26 total score, and Diet and Bulimia and food preoccupation subscales. However, multivariate analysis only confirmed the negative associations between social desirability and Diet and Bulimia and food preoccupation subscales. Higher social desirability had a positive association with Oral self-control, which was confirmed after considering other effects in the multivariate analysis. Given the lack of studies in the literature that help us understand these results, we hypothesize an interpretation based on how Oral self-control is perceived. Individuals may exhibit positive thinking of Oral self-control, while aspects that are assessed by the other subscales will tend to be more consistently perceived as negative. In addition, the questions on the Oral self-control subscale mainly revolve around the subjects’ perception of other people’s reactions and the subject’s opinion of how they actually eat food [40]. It should be noted that no studies have been found that relate social desirability to the risk of eating disorders, which leads to the need for further studies assessing this potential source of bias.

This study has some limitations related to the sample’s representativeness, namely, the overall low proportion of participants considering the total students contacted, the sample sizes of the distinct course areas, and the small number of male nutrition student participants. Despite these limitations, the absence of research focusing on the possible effects of social desirability on the risk assessment of eating disorders in nutrition/dietetics students and other areas evidences the important contribution of the present study to this area of knowledge. It is worth noting that the small number of male nutrition students is in line with their proportion on this course.

Future research should primarily address these limitations. Furthermore, understanding the impact of increased knowledge, through the analysis of academic year, on the risk of eating disorders is important, as it may have implications for the professional practice of these future health professionals, and it may be necessary to create interventions among nutrition students aimed at reducing the possible effects.

## 5. Conclusions

Our study found no differences in the risk of eating disorders between nutrition and dietetics students and students from other courses, neither between different academic years. A high risk of eating disorders was significantly more prevalent among female students. Social desirability showed a negative correlation with the Diet and Bulimia and food preoccupation subscales and a positive correlation with Oral self-control. Therefore, it should be considered when analyzing the EAT-26 subscales to assess the risk of eating disorders.

## Figures and Tables

**Table 1:** Age, BMI, EAT-26 scale and subscales, and social desirability: participants’ characteristics and sex comparison.

	Total (n = 799)	Females (n = 653)	Males (n = 146)	*p* *
	Mean (SD)	Mean (SD)	Mean (SD)	
Age (years)	21.2 (2.5)	21.0 (2.5)	21.8 (2.6)	0.002
Adjusted BMI (kg/m^2^)	22.6 (3.8)	22.4 (3.7)	23.2 (4.0)	0.023
Desired BMI (kg/m^2^)	21.4 (2.2)	21.0 (2.0)	22.7 (2.5)	<0.001
Wished BMI change (kg/m^2^)	0.9 (2.5)	1.1 (2.5)	0.1 (2.6)	<0.001
EAT-26				
Total score (range: 0 to 78)	9.8 (9.8)	10.2 (10.2)	7.9 (7.0)	0.059
Diet (range: 0 to 39)	4.8 (6.5)	5.1 (6.8)	3.6 (4.6)	0.063
Bulimia and food preoccupation (range: 0 to 18)	2.0 (2.7)	2.1 (2.9)	1.8 (2.2)	0.576
Oral self-control (range: 0 to 21)	2.9 (3.0)	3.0 (3.1)	2.5 (2.6)	0.140
Social desirability (range: 0 to 13)	6.7 (2.6)	6.7 (2.5)	6.9 (3.0)	0.549

* Comparison between sexes (independent samples Student’s *t* test). BMI: body mass index. EAT-26: Eating Attitudes Test-26. SD: standard deviation.

**Table 2:** Comparison between courses regarding age, BMI, EAT-26 scale and subscales, and social desirability.

	Nutrition and Dietetics (n = 110)	Other Areas of Human Health (n = 202)	Other Non-Health-Related Areas (n = 487)	*p* *
	Mean (SD)	Mean (SD)	Mean (SD)	
Age (years)	21.1 (2.5)	21.2 (2.5)	21.2 (2.5)	0.828
Adjusted BMI (kg/m^2^)	21.8 (2.6)	22.4 (3.2)	22.8 (4.2)	0.160
Desired BMI (kg/m^2^)	21.1 (1.9)	21.3 (1.9)	21.4 (2.3)	0.558
Wished BMI change (kg/m^2^)	0.5 (1.4)	0.8 (2.2)	1.1 (2.8)	0.373
EAT-26				
Total score (range: 0 to 78)	8.9 (8.0)	9.7 (9.5)	10.0 (10.2)	0.930
Diet (range: 0 to 39)	4.3 (5.0)	5.0 (6.6)	4.9 (6.8)	0.872
Bulimia and food preoccupation (range: 0 to 18)	1.7 (2.5)	2.0 (2.6)	2.2 (2.9)	0.209
Oral self-control (range: 0 to 21)	3.0 (3.2)	2.8 (2.6)	3.0 (3.1)	0.945
Social desirability (range: 0 to 13)	6.9 (2.6)	7.0 (2.7)	6.6 (2.5)	0.155

* Comparison between courses (one-way ANOVA). BMI: body mass index. EAT-26: Eating Attitudes Test-26. SD: standard deviation.

**Table 3:** Correlations between the EAT-26 total and subscales and academic year, age, adjusted and wished BMI change, and social desirability.

	Bulimia and Food Preoccupation	Oral Self-Control	EAT-26	Academic Year	Age	Adjusted BMI	Wished BMI Change	Social Desirability
Diet	0.594 (<0.001)	0.080 (0.024)	0.823 (<0.001)	−0.021 (0.558)	−0.005 (0.893)	0.296 (<0.001)	0.412 (<0.001)	−0.129 (<0.001)
Bulimia and food preoccupation		0.055 (0.121)	0.699 (<0.001)	−0.052 (0.141)	−0.064 (0.070)	0.179 (<0.001)	0.267 (<0.001)	−0.180 (<0.001)
Oral self-control			0.488 (<0.001)	−0.071 (0.046)	−0.112 (0.001)	−0.281 (<0.001)	−0.261 (<0.001)	0.139 (<0.001)
EAT-26				−0.076 (0.031)	−0.080 (0.025)	0.111 (0.002)	0.212 (<0.001)	−0.080 (0.024)
Academic year					0.446 (<0.001)	0.040 (0.246)	0.039 (0.270)	−0.016 (0.656)
Age						0.153 (<0.001)	0.110 (0.002)	0.011 (0.746)
Adjusted BMI							0.730 (<0.001)	−0.107 (0.002)
Wished BMI change								−0.161 (<0.001)

Sample size: n = 799. Values expressed as Pearson’s correlation coefficient (*p*). The transformed variables of adjusted BMI and wished BMI change and EAT-26 total score and subscales were used in the analysis. EAT-26: Eating Attitudes Test-26. BMI: body mass index.

**Table 4:** Effects of sex, age, course, academic year, BMI, and social desirability on the EAT-26 total and subscales.

	n	Diet			Bulimia and Food Preoccupation			Oral Self-Control			EAT-26		
		Beta	*p*	*η_p_* *^2^*	Beta	*p*	*η_p_* *^2^*	Beta	*p*	*η_p_* *^2^*	Beta	*p*	*η_p_* *^2^*
Corrected model	799		<0.001	0.180		<0.001	0.104		<0.001	0.107		<0.001	0.063
Sex	799												
Female	653	−0.381	0.193	0.002	−0.219	0.133	0.003	0.412	0.057	0.005	−0.285	0.631	0.000
Male	146	0 (Ref.)			0 (Ref.)			0 (Ref.)			0 (Ref.)		
Age	799	−0.051	0.268	0.002	−0.049	0.034	0.006	−0.048	0.157	0.003	−0.175	0.061	0.004
Course	799		0.302	0.003		0.517	0.002		0.485	0.002		0.890	0.000
Nutrition and dietetics	110	0.406	0.187	0.002	−0.170	0.266	0.002	−0.263	0.247	0.002	−0.023	0.970	0.000
Other areas of human health	202	0.276	0.269	0.002	0.000	0.997	0.000	−0.110	0.550	0.000	0.231	0.647	0.000
Other non-health-related areas	487	0 (Ref.)			0 (Ref.)			0 (Ref.)			0 (Ref.)		
Academic year	799	−0.060	0.458	0.000	−0.031	0.444	0.001	−0.044	0.470	0.001	−0.230	0.163	0.002
Adjusted BMI	799	−0.022	0.668	0.000	−0.023	0.366	0.001	−0.111	0.003	0.011	−0.171	0.099	0.003
Wished BMI change	799	1.019	<0.001	0.082	0.335	<0.001	0.037	−0.234	0.009	0.009	1.262	<0.001	0.032
Social desirability	799	−0.081	0.044	0.005	−0.080	<0.001	0.020	0.090	0.003	0.011	−0.104	0.202	0.002

Multivariate ANalysis Of VAriance (MANOVA, except for EAT-26, where UniANOVA was used). The transformed variables of adjusted BMI and wished BMI change and EAT-26 total score and subscales were used in the analysis. BMI: body mass index. EAT-26: Eating Attitudes Test-26.

## Data Availability

Data are available upon request from the corresponding author, B.M.P.M.O.
